# The Road towards Polyclonal Anti-SARS-CoV-2 Immunoglobulins (Hyperimmune Serum) for Passive Immunization in COVID-19

**DOI:** 10.3390/life11020144

**Published:** 2021-02-15

**Authors:** Daniele Focosi, Marco Tuccori, Massimo Franchini

**Affiliations:** 1 North-Western Tuscany Blood Bank, Pisa University Hospital, 56124 Pisa, Italy; 2 Division of Pharmacology and Pharmacovigilance, University of Pisa, 56126 Pisa, Italy; marco.tuccori@gmail.com; 3 Unit of Adverse Drug reaction Monitoring, Pisa University Hospital, 56124 Pisa, Italy; 4 Department of Hematology and Transfusion Medicine, Carlo Poma Hospital, 46100 Mantua, Italy; massimo.franchini@asst-mantova.it

**Keywords:** COVID-19, SARS-CoV-2, polyclonal immunoglobulins, convalescent plasma, Cohn’s fractionation, IgG, passive immunotherapy

## Abstract

Effective treatments specific for COVID-19 are still lacking. In the setting of passive immunotherapies based on neutralizing antibodies (nAbs), randomized controlled trials of COVID-19 convalescent plasma (CCP) anti-SARS-CoV-2 Spike protein monoclonal antibodies (mAb), which have been granted emergency use authorization, have suggested benefit in early disease course (less than 72 hours from symptoms and seronegative). Meanwhile, polyclonal immunoglobulins (i.e., hyperimmune serum), derived either from CCP donations or from animals immunized with SARS-CoV-2 antigens, are likely to become the next nAb-derived candidate. We here discuss the pros and cons of hyperimmune serum versus CCP and mAb, and summarize the ongoing clinical trials of COVID-19 hyperimmune sera.

## 1. Introduction

The ongoing COVID-19 pandemic caused by SARS-CoV-2 is totaling more than 101 million cases and 2.1 million deaths worldwide as of 1 February 2021. Many prophylactic and therapeutic regimens have been tested in randomized controlled trials (RCT), but to date, only dexamethasone (6 mg once daily) [1] and remdesivir [2] have shown clear evidence of clinical benefit. Several vaccines are likely to make an impact on the pandemic [3], but herd immunity is far from being achieved in most countries.

Many hopes rely on passive immunotherapies based on anti-Spike neutralizing antibodies (nAbs), which develop after infection in close to 90% of patients and persist for at least 5 months [4]. There have been three different exploitations of nAbs. The most immediate has been COVID-19 convalescent plasma (CCP) [5], whose efficacy seems promising in the early disease course [6] but for which many randomized controlled trials are still pending [7]. Antiviral monoclonal nAbs have also entered the market for emergency use authorization (EUA) [8]. Meanwhile, COVID-19 polyclonal IgG formulations (i.e., hyperimmune serum) are expected to be far cheaper than antiviral monoclonal nAbs (albeit with a lower mean affinity), and pharmacoeconomic benefits can make a huge difference in a pandemic setting. 

Several countries have begun to direct CCP donations towards plasma manufacturers [9], to investigate CCP fractionation resulting in polyclonal IgG formulations. Polyclonal IgG can be administered intravenously (IVIg), intramuscularly or, more rarely, intranasally. IVIg manufactured from CCP should not be confused with IVIg from nonconvalescent donors, which have shown some benefit at high doses as an immunosuppressive treatment for COVID-19 [10,11,12].

An alternative source of hyperimmune serum is the immunization of animals: this has been successfully used for decades to manufacture sera used in oncohematology and transplant patients. Large mammals have historically been the preferred animals (e.g., rabbit- or horse-derived anti-thymocyte globulins [13]), but hen egg yolk containing IgY is currently a promising platform [14,15].

Hyperimmune IVIg are typically prepared from pools of 100–1000 liters of plasma, and the timely creation of dedicated CCP production chains poses difficult GMP issues within plasma vendor plants [16,17]. In order to be economically sustainable, contract (privately run) fractionation typically requires well over 10,000 liters of plasma per year. On the other hand, domestic (state-owned) fractionation typically requires over 100,000–200,000 liters per year in addition to starting up a fractionation facility. An “on-the-bench” mini-pool fractionation scale (MPFS) process (5–10 liters of plasma, i.e., approximately 20 recovered CCP units) using disposable devices and based on caprylic acid precipitation has been under development in Egypt since 2003, and has proven effective at purifying immunoglobulins (six-fold enrichment) [18]: such a solvent/detergent (S/D) Virus Inactivation Bag Cascade (VIPS SA, Colombier, Switzerland) is currently under investigation in NCT04383548. Another method based on the Gradiflow™ electrophoresis-based separation technology is under development [19].

In this manuscript, we systematically review the ongoing clinical trials of COVID-19 hyperimmune serum.

## 2. Methods

On 31 January 2021 we searched, online, the World Health Organization International Clinical Trial Registry Platform (ICTRP) databases (accessed at https://www.who.int/docs/default-source/coronaviruse/covid-19-trials.xls), NIH ClinicalTrials database (accessed at www.clinicaltrials.gov), and Cytel Global Coronavirus COVID-19 Clinical Trial Tracker (accessed at www.covid-trials.org) using the following query: “Condition = Covid-19” AND “Other terms = immunoglobulin OR serum” AND “Study type = Interventional”. The 565 search results were individually screened to exclude nonrelevant studies.

## 3. Results

We identified 16 clinical trials employing polyclonal immunoglobulins from convalescent donors or immunized animals. The results are summarized in Table 1. 

A single trial investigated polyclonal immunoglobulins directly recovered from single donors via either immunoadsorption or double filtration plasmapheresis (DFPP), respectively: both procedures are typically therapeutic (meaning the subtracted volume is discarded rather than used for therapeutic purposes), and hence, the regulatory framework for this kind of hyperimmune serum is still to be defined. Conventional, pooled hyperimmune serum is instead under investigation in five (when sourced from immunized animals) and nine trials (when sourced from human CCP donations). 

In the current regulatory framework, human hyperimmune sera are likely to enter the market faster than animal-derived sera. Among the human hyperimmune sera, two formulations are being investigated in RCTs using CCP in the control arm (NCT04381858 and NCT04395170), which represents the current gold standard for nAbs. RCTs comparing hyperimmune serum to neutralizing mAbs are anticipated in the near future. The typical dose is in the range of 0.1 to 0.4 grams per kilogram of recipient body weight (with a single trial using a fixed dose of 4 g), with the dose repeated for 1 to 3 days.

The typical exclusion criteria for hyperimmune serum trials include patients with prior receipt of standard IVIg (not hyperimmune to SARS-CoV-2) within 45 days; a history of allergy to IVIg or plasma products; a history of selective IgA deficiency (<7 mg/dL) with a documented presence of anti-IgA antibodies; any medical conditions for which receipt of the required volume of intravenous fluid may be dangerous to the patient (including New York Association Class III or IV stage heart failure); and any of the following thrombotic or procoagulant disorders: acute coronary syndromes, cerebrovascular syndromes and pulmonary or deep venous thrombosis within 28 days of randomization, a history of the prothrombin gene mutation 20210, homozygous Factor V Leiden mutations, antithrombin III deficiency, protein C deficiency, protein S deficiency or antiphospholipid syndrome.

## 4. Discussion

Antiviral hyperimmune sera are already marketed for the post-exposure prophylaxis of hepatitis B (hepatitis B immune globulin (HBIG), targeting hepatitis B surface antigen—HbsAg) [26], rabies virus [27] and, more interestingly, for another respiratory pathogen, respiratory syncytial virus (RSV) [28,29]. Post-exposure prophylaxis (especially of healthcare workers), as well as the early treatment of cases, will likely remain the main indications for COVID-19 hyperimmune serum.

Plasma fractionation results in IgG purification, hence removing undesirable plasma components (e.g., prothrombotic factors) but also potentially depleting beneficial ingredients (e.g., several IgG subclasses, IgA, antithrombin III, etc.). Additionally, most of the anti-SARS-CoV-2 neutralizing antibody responses in the IgG class have been shown to be associated with the IgG_1_ and IgG_3_ subclasses [30]: unfortunately, the IgG_3_ fraction is often depleted during industrial fractionation [31], and its impact on neutralizing titers should be carefully evaluated.

COVID-19 hyperimmune serum is considered a virally safe product since SARS-CoV-2 is inactivated by S/D treatment [32] and nonenveloped viral contaminants are additionally removed by nanofiltration. Fatty acids are also a good option for pathogen inactivation: in 2002, it was reported that caprylic acid [33] and octanoic acid [34] were as effective as S/D treatment at inactivating enveloped viruses.

In April 2020, the main plasma manufacturers (Takeda, Grifols [20], Biotest, CSL Behring, LFB, Octapharma, ADMA Biologics, BioPharma Plasma, GC Pharma, BPL, Sanquin, Liminal BioSciences, NBI, and Silanes) joined, instead, under The CoVIg19 Plasma Alliance (https://www.covig-19plasmaalliance.org/ (accessed on 11 February 2021)). The Bill & Melinda Gates Foundation is providing advisory support. Microsoft is providing technology including the Alliance website and the Plasma Bot, a self-screening tool that anyone can use to see if they qualify to donate their plasma. In October 2020, Takeda, which initially led the Alliance, withdrew the phase 1b RCT of its TAK-671 hyperimmune serum formulation (NCT04464460) as a business decision, focusing instead on the shared NCT04546581.

On the other side of the coin, lone players include Aegros Covimmune™ (https://aegros.com.au/ (accessed on 11 February 2021)), Lifefactors Zona Franca S.A.S. (https://lifefactors.com.co/ (accessed on 11 February 2021)), and the joint venture between the Italian Kedrion Biopharma S.p.a. and the Israeli Kamada Ltd, in collaboration with the Irving Medical Center of Columbia University [22].

Animal-derived hyperimmune serum is also worthy of investigation because it can be easily scaled up. Preliminary reports showed that the receptor binding domain (RBD) from the viral Spike glycoprotein elicits high titers of nAb in horses [23,35] and glycoengineered swine [36], and several companies embarked on clinical trials. Manufacturing animal sera often relies on different and/or additional steps when compared to human serum: for example, in order to avoid xenogeneic immunizations and serum sickness, plasma can be digested with pepsin under controlled conditions, rendering F(ab´)2 fragments from the immunoglobulin molecules. The fragments are then further purified by salting out and membrane Q chromatography. The bulk solution is typically nanofiltered (20 nm) and then sterilized through a 0.2 µm-pore cartridge.

Since plasma-derived drugs could theoretically suffer from many problems, including low potency, impurities (including infectious viruses [37] and clotting factors [38]), constraints on supply, the antibody-dependent enhancement (ADE) of infection, and batch-to-batch variation [39], polyvalent, 10^3^- to 10^4^-diverse recombinant hyperimmune antibody drugs generated from convalescent human blood donors, vaccinated human blood donors, and humanized animal repertoires will likely be an additional field of research for the coming years [40].

## 5. Conclusions

While the efficacy of COVID-19 hyperimmune serum remains hard to guess against a respiratory pathogen and in a landscape with quickly evolving viral strains, hyperimmune serum has several advantages over CCP (e.g., a smaller reinfusion volume, an easier administration route and easier preservation) and mAbs (more diversified nAbs against emerging variants of concern, and far cheaper), summarized in Table 2, making it a product worth being fully investigated in clinical trials. A few studies will be completed soon, and the results will drive the design of the next round of trials.

## Figures and Tables

**Table 1:** Summary or polyclonal immunoglobulin formulations under investigation for prevention or treatment of COVID-19, listed in World Health Organization International Clinical Trial Registry Platform (ICTRP) databases (accessed online at https://www.who.int/docs/default-source/coronaviruse/covid-19-trials.xls on 30 January 2021), NIH ClinicalTrials database (accessed online at www.clinicaltrials.gov on 30 January 2021), and Cytel Global Coronavirus COVID-19 Clinical Trial Tracker (accessed online at www.covid-trials.org on 30 January 2021). BSC: best supportive care; NA: not available.

Source	Recovery Method	Phase	NCT Identifier	Country	*n*	Start Date	Estimated Completion Date	Immunogen	Formulation (Brand Name)	Dosage	Indications
Convalescent humans	Immunoadsorption	I (vs. nonconvalescent IVIg)	NCT04264858	China	10	Mar 2020	May 2020	Whole virus	Full-length IgG	0.2 g/kg/day for 3 days	Treatment of severe COVID-19
	Double filtration plasmapheresis (DFPP)	I	NCT04418531	Italy	10	Jun 2020	Sep 2020	Whole virus	Full-length IgG	n.a.	Treatment of moderate COVID-19
	Plasma fractionation	III	NCT04546581	USA	500	Oct 2020	Jul 2021	Whole virus	Full-length IgG (H-Ig; CoVIg-19 Plasma Alliance)	n.a., single infusion	Treatment of moderate COVID-19 [20]
		I	NCT04548557	Pakistan	60	Sep 2020	Nov 2020	Whole virus	Full-length IgG	4 dose-finding arms of 0.15 to 0.3 g/kg	Treatment of severe and critically ill COVID-19
		II	NCT04383548	Egypt	100	Jun 2020	Jan 2021	Whole virus	Full-length IgG	Prophylaxis in younger than 20 years	Post-exposure prevention (group A) and treatment of moderate COVID-19 (group B)
		I/II (vs. BSC)	NCT04521309	Pakistan	50	Jun 2020	Mar 2021	Whole virus	Full-length IgG	4 dose-finding arms of 0.2 to 0.35 g/kg	Treatment of severe and critically ill [21]
		II (vs. BSC)	NCT04555148	South Korea	60	Sep 20	Aug 2021	Whole virus	Full-length IgG (GC5131)	3 dose-finding arms	Treatment of moderate COVID-19
		I/II	NCT04550325	Israel	12	Aug 2020	Nov 2020	Whole virus	Full-length IgG	4 g	Treatment of mild COVID-19 [22]
		III (vs. CCP)	NCT04381858	Mexico	500	May 2020	Nov 2020	Whole virus	Full-length IgG	0.3 gr/kg/day (5 doses) vs. CCP > 1:640	Treatment of severe COVID-19
			NCT04395170	Colombia	75	Sep 2020	Jun 2021	Whole virus	Full-length IgG (Life Factor Zona Franca S.a.s.)	0.1 g/kg (max 50 g) on days 1 and 3	Treatment of moderate COVID-19
Bovine		Ib (vs. placebo)	NCT04469179	USA	21	Sep 2020	Dec 2020	Anti-wild-type and recombinant S from insect cells	Full-length IgG (SAB-185)	3 dose-finding arms of 10 to 50 mg/kg	Treatment of COVID-19 outpatients
Equine		I/II	NCT04573855	Brazil	41	Dec 2020	Mar 2021	n.a.	F(ab’)_2_	n.a.	Treatment of moderate COVID-19 [23]
		II	NCT04610502	Costa Rica	26	Sep 2020	Dec 2020	Anti-Santi-S1,N,S1+E+M	Full-length IgG	1 10 mL vial on day 1	Treatment of moderate and severe COVID-19 [24]
		II/III	NCT04494984	Argentina	242	Jul 2020	Dec 2020	RBD	F(ab’)_2_ (INM05; Inmunova s.a.)	4 mg/kg on days 1 and 3; mean PRNT 1:10,240.	Treatment of early, moderate to severe COVID-19 [25]
		I/II (vs. placebo)	NCT04514302	Mexico	51	Oct 2020	Jun 2021	n.a.	F(ab’)2 (INOSARS)	2 dose-finding arms (2 vials and 6 vials)	Treatment of moderate COVID-19
Hen		I (vs. placebo)	NCT04567810	Australia	48	Sep 2020	Dec 2020	n.a.	IgY	6 dose-finding arms (2 to 24 mg total dose)	Healthy subjects [15]

**Table 2:** A comparison of neutralizing antibody (nAb)-based therapies for COVID-19.

	CCP	Hyperimmune Serum (Polyclonal IgG)	Monoclonal Antibodies
Speed of access	Weeks (as soon as convalescents appear)	>1 year	>1 year
Safety issues	Safe (pathogen inactivation, possible plasma protein allergies)	Safe (solvent/detergent, but ABO-incompatible)	Extremely safe (recombinant technology)
Potency	Very high (high PRNT titer; includes neutralizing IgA and IgM, and factors other than antibodies)	High (no IgA; less IgG_3_)	High (nanomolar IC_50_) Very high for Ab cocktails
Cost	€	€€	€€€€
Logistics	+2–+8°C (if fresh) or <−25°C (if frozen); i.v.	+2–+8°C; s.c./i.v.	+2–+8°C; s.c./i.v.
Scalability	Not easily scalable	Easily scalable	Very easily scalable

PRNT, plaque reduction neutralization test.

## Data Availability

Please refer to suggested Data Availability Statements in section “MDPI Research Data Policies” at https://www.mdpi.com/ethics (accessed on 11 February 2021).
